# Tissue Carcinoembryonic Antigen, Calcium, Copper and Iron Levels in Cancerous Lung Patients

**DOI:** 10.3779/j.issn.1009-3419.2011.01.06

**Published:** 2011-01-20

**Authors:** Yousuf ALWAHAIBI Nasar, Sultan ALGHARIBI Jokha, Salim ALSHUKILI Amna, Khalifa ALSHUKILI Ahmed

**Affiliations:** 1 Department of Pathology, College of Medicine and Health Sciences, Sultan Qaboos University, Muscat, Oman; 2 Department of Microbiology and Immunology, College of Medicine and Health Sciences, Sultan Qaboos University, Muscat, Oman

**Keywords:** Calcium, Copper, Iron, Human CEA protein, Immunohistochemistry, Lung neoplasms

## Abstract

**Background and objective:**

The expression of various trace elements and markers in lung cancer is controversial. The aim of this study is to evaluate the presence of calcium (Ca), copper (Cu), iron (Fe) and carcinoembryonic antigen (CEA) in cancerous untreated lung tissues and to determine a possible association between these markers and lung cancer.

**Methods:**

Fourty-eight cancerous lung tissue blocks, from Sultan Qaboos University Hospital, Sultanate of Oman, were studied. Fe, Ca, Cu, and CEA were demonstrated in the tissue blocks using Perl's Prussian blue, Von Kossa's, modified rhodanine and immunohistochemical staining methods, respectively.

**Results:**

Twenty-three of 48 specimens showed positive Fe staining, 2 showed positive Ca staining and Cu was absent in all specimens. 93.7% expressed CEA in varying degree of positivity. 81.25% of these sections showed high expression of CEA.

**Conclusion:**

Tissue concentrations of trace elements were not elevated in lung cancer and therefore cannot be considered as a potential marker. Despite the low sensitivity and specificity of CEA as previously reported, tissue CEA should be considered as a potential marker in the evaluation of lung cancer.

## Introduction

Lung cancer remains the most common cause of cancer related death in Europe and in the United States and it accounts for 1.2 million new cases annually^[1]^. The exact cause of lung cancer is unknown. However, long term exposure to tobacco smoke is one of the main factors of lung cancer. In addition, there are other causes in non-smokers and account for as many as 25% of lung cancer cases such as genetic factors, radon gas, asbestos, viruses and particulate matter in air pollution^[2]^. The available methods that are used to cure lung cancer would be more successful if the tumor could be detected earlier. The research continues for selecting certain markers that would give a significant value in the diagnosis and management of lung cancer and other diseases.

In recent years, trace elements have received considerable attention in relation to the formation of many diseases including cancer. Some of the trace elements play an important role in normal physiological process and also in various diseases^[3-5]^. In fact, several diseases are linked to the toxicity or deficiency of some of the trace elements. For example, iron deficiency anemia, Rickets and Keshan diseases are due to the deficiency of iron, calcium and selenium, respectively.

Carcinoembryonic antigen (CEA) is a glycoprotein containing 50% carbohydrates with a molecular weight of 200 kDa^[6]^. The human *CEA* gene family contains 29 different genes, which have various functions and expressed normally in different cells and tissues such as pyloric mucous cells, colon, liver, kidney, urinary bladder and others^[7, 8]^. Furthermore, high levels of CEA can be seen in various cancers such as colorectal, breast, pancreatic, gallbladder, hepatocellular and thyroid carcinomas^[9-11]^. In addition, nonmalignant diseases such as colitis and emphysema have also high CEA expression^[12]^.

Not many studies have investigated the expression of CEA in cancerous lung tissues. This could be due to CEA's low sensitivity and specificity as a tumor marker in tissues^[13]^. In addition, the joint statement of European Respiratory Society and the American Thoracic Society on serum lung cancer did not include CEA or any other markers for diagnosis, screening, staging or monitoring the effects of cancer treatment^[14]^. The aim of this study is to evaluate the presence of Ca, Cu, Fe and CEA in cancerous untreated lung tissues and to determine a possible association between these markers and lung cancer.

## Materials and methods

### Paraffin blocks

Paraffin blocks containing paraffin-embedded specimens of confirmed untreated lung cancer were taken from the archival files of the Department of Pathology of Sultan Qaboos University Hospital, from 1992 to 2009. Seventynine cases of lung cancers were found and 31 blocks were excluded because some of them could not be seen while others had insufficient amount of tissues and so 48 blocks were obtained. The blocks were cut into sections of 3 μm thickness using a rotary microtome. Ethical Approval was obtained from the Medical Research Committee and Ethics Committee (MREC) from College of Medicine and Health Sciences, Sultan Qaboos University, Sultanate of Oman (MREC # 341).

### Histochemical staining

Perl's Prussian blue, Von Kossa's and modified Rhodanine methods were used to stain iron, calcium and copper, respectively^[15]^. Known positive controls were treated as the test.

### CEA staining

Paraffin sections were dewaxed in xylene (three changes), hydrated through graded concentrations of alcohol (100%, 95%, 70% and 50%) and then to water for 5 min. After the sections were washed 3 times, 15 min each, with Tris buffered saline (TBS), they were incubated with 3% H_2_O_2_ for 30 min at room temperature to block endogenous peroxidase activities. The sections were then washed 3 times for 15 min each with TBS. The sections were incubated with CEA (Dako, Denmark, Code No A0115) 1:1, 000 diluted in TBS at room temperature for 30 minutes. After washing 3 times, 15 min each with TBS, the sections were covered with Envision horseradish peroxidase (HRP) labeled polymer (Dako, Denmark, Code No K4061) and incubated at room temperature for 30 min. After washing 3 times, 15 min each, with TBS, the sections were covered with 3'3-diaminobenzidine (DAB) solution for 3 min. After washing in running tap water for 5 min, the sections were counterstained with haematoxylin for 3 min and blued in running tap water for 5 min. Finally, the sections were dehydrated in alcohol (3 changes), cleared in xylene (three changes) and mounted in DPX. Known positive control and negative control (incubation of CEA was omitted) were used to confirm the specificity of CEA antibody.

### Assessment

Staining for Fe, Cu, and Ca was graded by subjective interpretation of light microscope finding. Positive iron staining was defined in the lung tissues by detecting blue pigmentation of intracellular or extracellular granules. Positive calcium staining was defined by detecting black deposits in the tissue, while positive copper staining was defined by detecting red to orange deposits. All were also matched against the control sections. All slides were reviewed by two investigators. The degree of staining was graded by the following criteria: grade (0): no detectable staining deposits, grade (1+): trace staining deposits, grade (2+): moderate to occasional number of staining deposits, grade (3+) abundant sta ining deposits^[16]^. Staining for CEA was graded by the following criteria: (-) = negative reaction, (+) = weak positive staining (< 5%), (++) = moderate positive staining (5%-50%), (+++) = strong positive staining (> 50%) ^[17]^.

## Results

### General features

There were 33 men (mean age 63±36) and 15 women (mean age 59±47), age range 16-81. The ratio of lung cancer in men to women was 2.2:1 (33:15). The cell types of the tumors included differentiated carcinoma (22), adenocarcinoma (10), non small cell carcinoma (8), small cell carcinoma (3), spindle cell tumor (3) and non-Hohgkin's lymphoma (2).

### Histochemical staining

Table 1 shows that among 48 cases of cancerous lung sections, 23 (47.9%) showed positive iron staining (Fig 1A), 2 (4.2%) showed positive calcium staining (Fig 1B), while no specimen showed positive copper staining (Fig 1C). However, not all the specimens that showed positive iron staining had the same grade. 39.6% showed trace iron granules, while only 4.2% showed moderate and 4.2% showed abundant iron granules. On the other hand, 52.1% of the cases were negative for iron staining.

**1:** Metal grades in cancerous lung sections

Grade	Cu	Ca	Fe
0	48 (100%)	46 (95.8%)	25 (52.0%)
1+	0	1 (2.1%)	19 (39.6%)
2+	0	1 (2.1%)	2 (4.2%)
3+	0	0	2 (4.2%)

**1:**
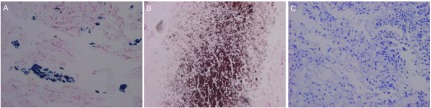
Iron (A), calcium (B), and copper (C) deposits in cancerous lung tissues. A: Iron deposits (blue with grade +++) in cancerous lung tissues (Perl's Prussian blue method, ×200); B: Calcium deposits (brown with grade ++) in cancerous lung tissues (Von Kossa's method, ×200); C: The absence of copper in all cancerous lung tissues (Modified Rhodanine method, ×200).

Comparison of Perl's Prussian blue, Von Kossa's and modified Rhodanine methods and H&E staining method of cancerous lung sections for all 48 cases showed that H&E method was not reliable and cannot be used as a method for the demonstration of Fe, Ca and Cu (Tab 2). There were 23 sections in which Perl's staining was positive but only 2 positive iron sections were seen on the H&E stained sections.

**2:** Comparison of H&E staining and Perl's Prussian blue, Von Kossa's and modified Rhodanine methods on cancerous lung sections

		H&E staining		Total
		Positive	Negative	
Perl's stain	Positive	2	21	23
	Negative	0	25	25
Von Kossa' stain	Positive	0	2	2
	Negative	0	46	46
Rhodanine stain	Positive	0	0	0
	Negative	0	48	48

### Immunohistochemical staining

The immunohistochemical demonstration of CEA showed that 45 of 48 cancerous lung sections were positive (Fig 2A). However, there was a considerable variation in positivity (Fig 2B). 21 of 45 positive cases showed more than 50% (+++) CEA staining while between 5%-50% (++) was observed in 18 cases. In addition, less than 5% (+) was observed in 6 of 45 positive cases. On the other hand, only 3 cases showed the absence of CEA expression.

**2:**
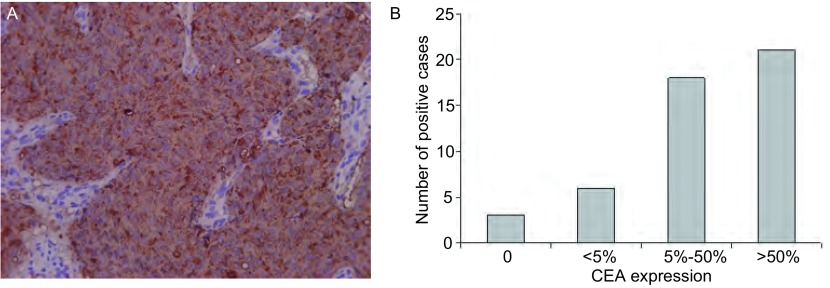
CEA expression in cancerous lung tissues. A: CEA expression in cancerous lung tissues (Immunohistochemical method, ×200); B: CEA expression in cancerous lung tissues.

## Discussion

Although CEA is used as a predictor of recurrence and indicator of poor prognosis for lung cancer patients^[18, 19]^, many studies have limited its uses in prognosis of lung cancer^[20, 21]^. In this study, the immunohistochemical staining of CEA showed a high expression in cancerous untreated lung tissues. Unfortunately, it was very difficult to obtain normal lung tissue from organ donors or even nonnewplastic tissues, to compare with the cancerous tissues. Immunohistochemical elevation of tissue CEA should not be examined alone as many factors such as extent of the disease, environmental pollution and chemo and radio therapies, may contribute to its findings. The finding of this study is on agreement with other previous study which reported that CEA was found in 90% of malignant pulmonary tumors^[22]^. In addition, other previous studies have reported that higher expression of serum CEA was found in patients with adenocarcinoma in comparison with those of squamous cell carcinoma^[13, 23]^. The major difference between the findings of this study and those of earlier studies is that this method evaluated the concentration of CEA on lung tissues rather than in serum fluids. Malignant cells release CEA into bloodstream and then start to deposit in various tissues^[24]^.At what stage of carcinogenesis, CEA is produced, remains to be known. Many studies have conflicting results on the level of CEA in normal and cancerous tissues.

The concentrations of trace elements are usually measured by particle induced X-ray emission (PIXE), atomic absorption spectrometry (AAS), neutron activation analysis (NAA), X-ray fluorescence analysis (XRF) or total reflection geometry method (TRXRF). However, in this study, Perl's Prussian blue, Von Kossa's and modified Rhodanine methods were used to stain iron, calcium and copper, respectively. Those methods are direct and specific and unlike with other methods, the chance of contamination is very less.

Iron, which is an essential mineral for many cellular processes, can be a source of pulmonary injury. When iron balance is not maintained, excessive accumulation of iron could activate free radicals and reactive oxygen species leading to the formation of oxidative stress^[25]^. Subsequently, ox idative stress can cause many pulmonar y diseases, including cancer^[26]^. In addition, the lungs are subject to various circulating metals in the atmosphere^[27]^. Iron is considered to be the greatest abundant metal in air pollution particles^[28]^. It has been calculated that a person breathing a volume of 500 mL and a respiratory rate of 200 μg per min is exposed to about 10 μg of iron every day^[28]^. In this study, elevated iron was observed in less than 50% cases and 39.6% of these cases showed only trace iron granules. This might indicate that cancerous lung tissues are not associated with the presence of iron metal.

Calcium occurs in the lung cancer by four possible mechanisms: calcified scar tissue or granulomatous disease engulfed by tumor, dystrophic calcification within areas of tumor necrosis, calcium deposition within the tumors as a result of a secretory function of the carcinoma and metastatic calcification in normal lung tissues^[29, 30]^. However, this study showed only 4.2% of all cases suggesting that calcification in cancerous lung tissues is not a common feature and does not correlate with the type of lung cancer. In addition, it is uncertain of the mechanism by which these two cases underwent calcification. The finding of the study disagrees with other previous study, which reported a higher level of calcium in malignant lung tissues^[31]^.

Copper level was absent in all cases, in agreement with this study, recent review on the measurement of copper in Wilson disease, which has a high level of copper, has shown that rhodanine histochemical method did not detect the total level of copper in liver tissues^[32]^. Although rhodanine method is specific for copper, its sensitivity needs to be investigated, in particular, with tiny amount of copper. On the other hand, several studies have shown that serum copper levels were elevated in patients with different diseases including cancer^[33, 34]^. In addition, copper levels have been found in patients with a late stage of disease, declined in patients responding to treatment and increased prior to relapse^[35]^.

Furthermore, in the evaluation of the efficiency of H&E staining method, Fe, Ca and Cu were examined using H&E method. H&E failed to stain those elements with the exception of iron, which showed in only two cases (abundant). Therefore, the examination of H&E sections to assess Fe, Ca and Cu metals cannot replace Perl's Prussian blue, Von Kossa's and modified Rhodanine methods, respectively.

As a limitation of this study, we should point out the lack of normal lung tissues for comparison with the cancerous tissues. The relatively small number of lung cancer blocks was another drawback. Thus future investigation should investigate the possible explanations for CEA increase in cancerous untreated lung tissues with the possibility of including normal lung tissues. In conclusion, Fe, Ca and Cu trace elements are less important in the investigation of lung cancer. Despite the low sensitivity and specificity of CEA as previously reported, tissue CEA should be considered as a potential marker in the evaluation of lung cancer.

## Acknowledgements

The authors wish to thank Mr. Johanes Selva Kumar for editing the manuscript.

## Conflict of Interest

The authors declare no conflict of interest.
